# Thermal imaging dataset from composite material academic samples inspected by pulsed thermography

**DOI:** 10.1016/j.dib.2020.106313

**Published:** 2020-09-14

**Authors:** Jorge Erazo-Aux, Humberto Loaiza-Correa, Andres David Restrepo-Giron, Clemente Ibarra-Castanedo, Xavier Maldague

**Affiliations:** a Escuela de Ingeniería Eléctrica y Electrónica, Universidad del Valle, Cali, VA 760032, Colombia; b Facultad de Ingeniería, Institución Universitaria Antonio José Camacho, Cali, VA 760046, Colombia; c Computer Vision and Systems Laboratory, Laval University, Quebec City, QC G1V 0A6, Canada

**Keywords:** Thermal imaging, Composite materials, Pulsed thermography, Non-destructive testing

## Abstract

This paper presents a thermal imaging dataset from composite material samples (carbon and glass fiber reinforced plastic) that were inspected by pulsed thermography with the goal of detecting and characterizing subsurface defective zones (Teflon inserts representing delaminations between plies). The pulsed thermography experiment was applied to 6 academic plates (inspected from both sides) all having the dimensions of 300 mm x 300 mm x 2 mm and same distribution of defects but made of different materials: three plates on carbon fiber-reinforced plastic (CFRP) and three plates made on glass fiber reinforced plastic (GFRP) specimens with three different geometries: planar, curved and trapezoidal. Each plate contains 25 inserts having length/depth ratios between 1.7 and 75. Two FX60 BALCAR photographic flashes (6.2 kJ per flash) were used to generate the heat pulse (2 ms duration), an X6900 FLIR infrared camera using ResearchIR software to record the thermal images and a custom-built software/control unit to synchronize data recording with pulse generation. Finally, the dataset proposed consists of 12 sequences of approximately 2000 images of 512 × 512 pixels each.

**Specifications Table**

**Table:** 

Subject	Materials Science
Specific subject area	Materials Science (General)
Type of data	Tables CSV (comma-separated values)
How data were acquired	Pulsed thermography (PT) (see Fig. 1) Instruments: Infrared camera (FLIR, Inc. X6900) [1] Photographic/Power flashes (BALCAR, Inc. FX60) Image acquisition software (FLIR, Inc. ResearchIR Max 4) [2] Data/pulse generation synchronization (custom-built software/hardware)
Data format	Raw data (.CSV files)
Parameters for data collection	Ambient temperature (21°C) Emissivity (0.9) Wind speed (0 m/s) Distance between IR camera and sample (100 cm) Angle between IR camera lenses and the sample surface (90°) Distance between flashes and sample (50 cm) Angle between flashes (each) and sample (45°). The heat pulses of them coinciding at the center of the sample surface.
Description of data collection	The infrared camera recorded the thermal evolution on the surface of the inspected CFRP/GFRP sample for several seconds (approximately 16 to 17 s) at 120 and 145 frames/s sampling rate, from before applying a heat pulse, during the heat pulse and during cooling. The experiment was performed from the two faces of specimen (providing shallow defect depths from the front face and deeper depths from the back face), and each sequence was labeled and saved into an independent file.
Data source location	Institution: Computer Vision and Systems Laboratory at Laval University City/Town/Region: Quebec City / Quebec / North America G1V 0A6 Country: Canada Latitude and longitude (and GPS coordinates) for collected samples/data: 46°46′45.4"N 71°16′34.7"W
Data accessibility	Data identification number: DOI: 10.17632/v4knrwgj9y.2 Direct URL to data: https://data.mendeley.com/datasets/v4knrwgj9y/2

## Value of the Data

• The research community can use the thermal images dataset to evaluate the infrared imaging processing techniques performance. The methods can focus on locating laminar defective regions and on the assessment of the attributes of these regions, such as the shape, size, depth, or specific materials properties of thermograms that compose the dataset.
• The research community can use the dataset of thermal images to develop and test processing strategies without having an experimental platform available.
• The dataset of raw thermal images contains undesirable effects of low contrast, non-uniform heating, and noise. It is possible to use the dataset to test or develop processing techniques to overcome these unwanted effects.
• Besides, the different features of the dataset (thermograms of two different materials, a variety of sample geometries, and defect sizes and locations) will allow a broader study concerning the scope and limitations of the processing methods applied to the images.

## 1 Data Description

The objective of the pulsed thermography experiment is to monitor the surface temperatures of the samples as a function of time and the flow of transient heat generated through an energy stimulus in the samples [3], [4], [5], [6]. The thermal stimulus allows the generation of enough temperature differences to identify sub-surface anomalies if they are present [7], [8], [9].

**Fig. 1:**
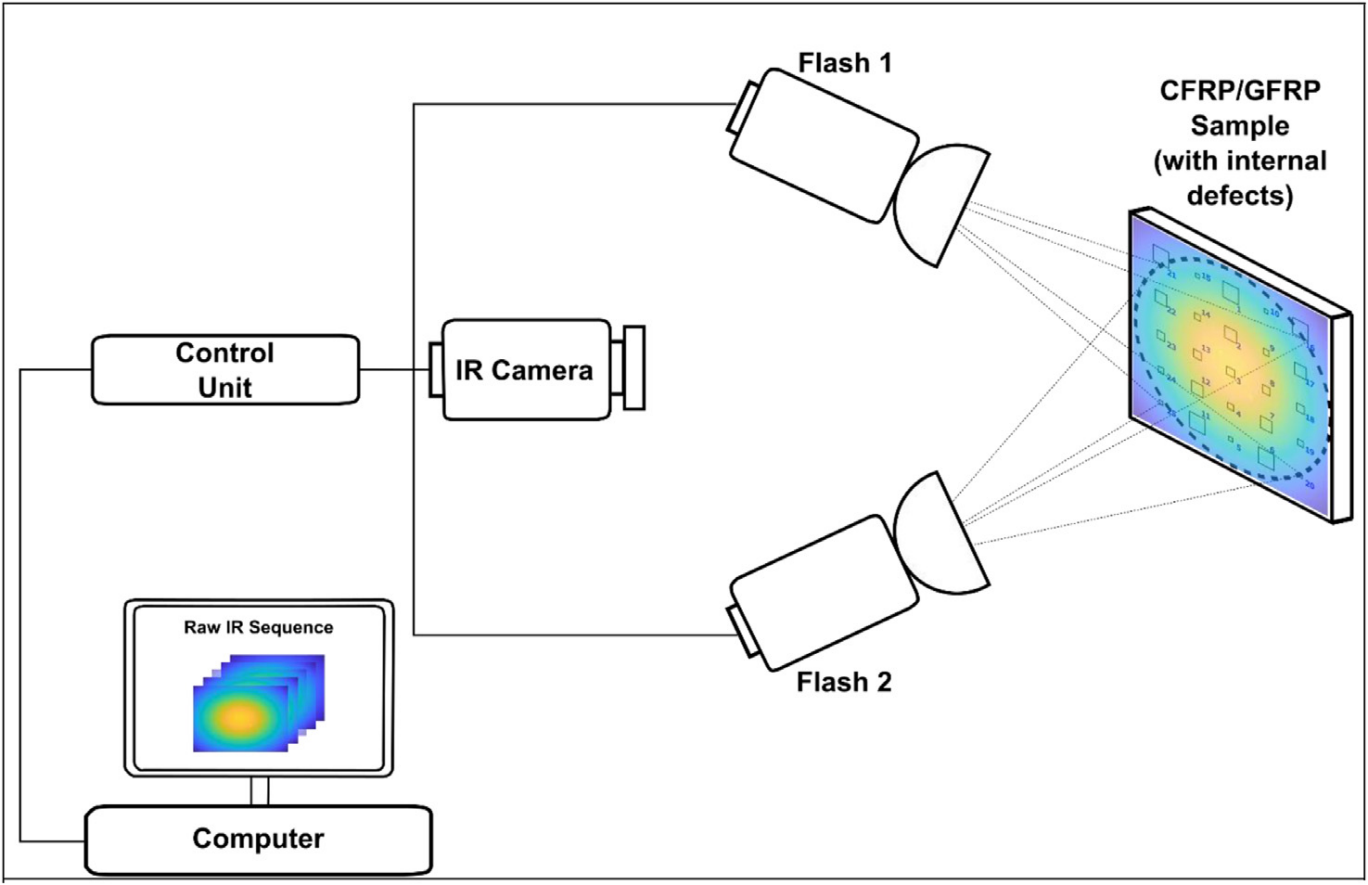
Pulsed thermography set up.

**Fig. 2:**
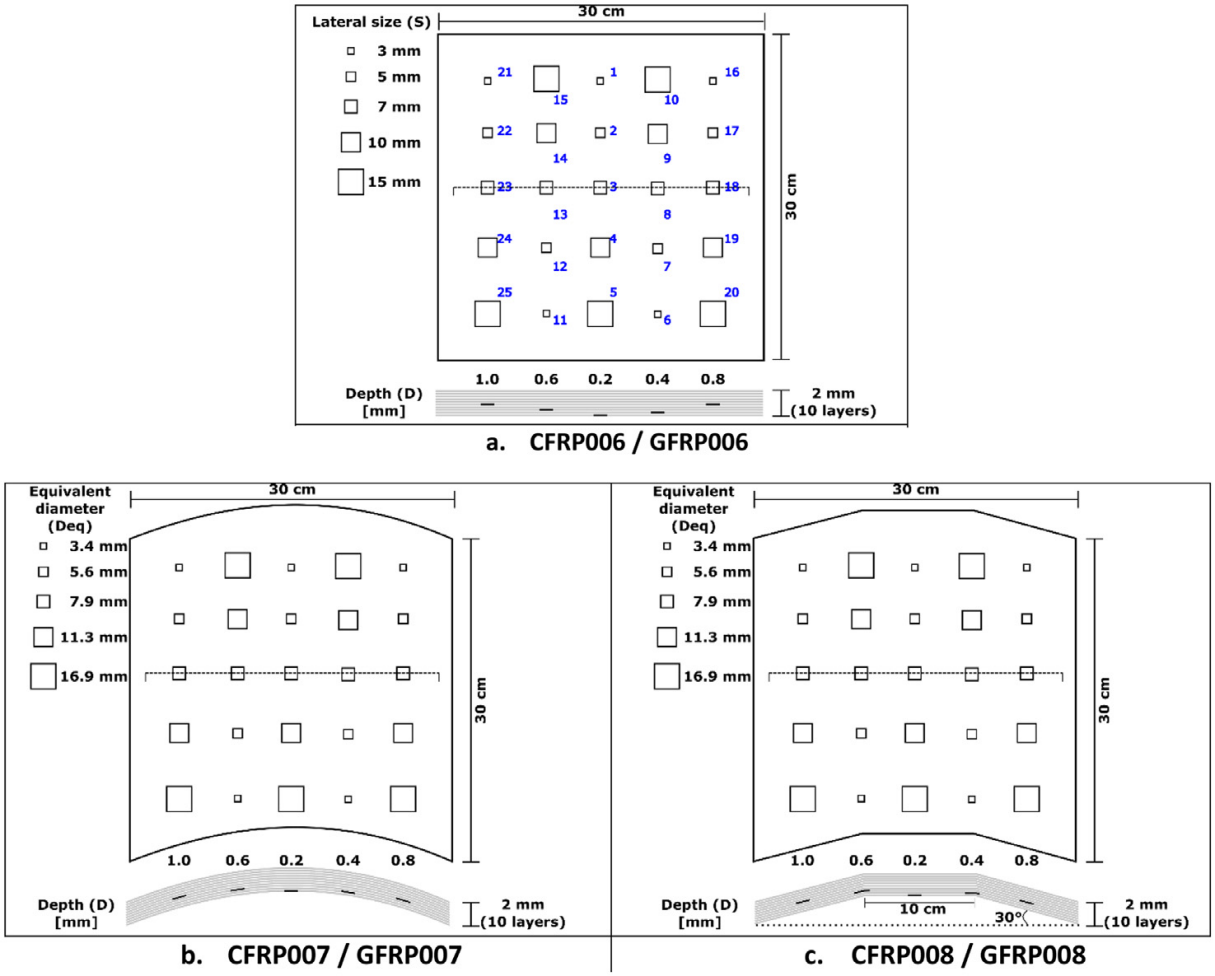
Geometry of CFRP and GFRP samples. Adapted from Ibarra-Castanedo [10].

The 12 image sequences provided in this dataset show the evolution of temperature over time on the surface of composite materials tested by pulsed thermography experiment (see Fig. 1). Sequences are stored in folders containing ‘.CSV’ files. Table 1 shows the physical properties of the materials. Table 2 presents the infrared camera specifications. Table 3 lists the acquisition conditions that were adjusted in the experiment. Fig. 2 shows the geometry of the CFRP/GFRP samples used and presents the characteristics of their defective zones. Lastly, Table 4 consolidates the dataset files information. Folders and files are label as CFRP/GFRP-**samplename**_facq-**frequencyvalue**_s-**sideofinspection**_Img-**frames**. This tagging scheme describes the sample name tested, the acquisition frequency used, the sample side of inspection taken, as well as the number of each frame (for .CSV files) within the sequence, and the whole number of frames from the image sequence (for the folders), respectively.

**Table 1:** Properties of used materials.

Material	Conductivity	Heat Capacity	Density
	[W/m.K]	[W.s/kg.K]	[kg/m^3^]
**CFRP (perpendicular to the surface)**	0.8	1200	1600
**GFRP (perpendicular to the surface)**	0.3	1200	1900
**Teflon**	0.25	1050	2170

**Table 2:** Infrared camera specifications.

Sensor	Image resolution	Intensity resolution	Acquisition frequency	Spectral range
	[pixels]	[bits]	[Hz]	[um]
**InSb** **CCD Matrix**	640 × 512 used: 512 × 512	14	0.0015 to 1004 Programmable 120 and 145 during tests	3.0 to 5.0

**Table 3:** Acquisition conditions for pulsed thermography experiment.

Sampling rate	Acquisition time (t_adq_)	Time step (Δt)	Truncation window w(t)	Total number of frames	
**[Hz]**	[s]	[s]	(s)	[-]	
**120**	> 16	0.0083	16	2000	
**Emissivity**	**Pulse width**	**Distance IR Camera – CFRP/GFRP sample**	**Angle IR Camera – CFRP/GFRP sample**	**Distance Flash – CFRP/GFRP sample**	Angle*Flashes – CFRP/GFRP sample
**[-]**	[ms]	[m]	[°]	[m]	[°]
0.98	2	1.0	90	0.5	45

⁎ The heat pulses of both flashes align in the center of the sample surface.

**Table 4:** Dataset files information.

Folder name	Acquisition frequency (f_adq_)	Specimen inspected	Side of inspection	Depths ranging
	[Hz]			[mm]
**CFRP-006_facq-145Hz _s-Front_Img-2000**	145	CFRP006	front	0.2 to 1
**CFRP-006_facq-120Hz _s-Back_Img-2000**	120	CFRP006	back	1 to 1.8
**CFRP-007_facq-145Hz _s-Front_Img-2000**	145	CFRP007	front	0.2 to 1
**CFRP-007_facq-120Hz _s-Back_Img-2000**	120	CFRP007	back	1 to 1.8
**CFRP-008_facq-145Hz _s-Front_Img-2000**	145	CFRP008	front	0.2 to 1
**CFRP-008_facq-120Hz _s-Back_Img-2000**	120	CFRP008	back	1 to 1.8
**GFRP-006_facq-145Hz _s-Front_Img-2000**	145	GFRP006	front	0.2 to 1
**GFRP-006_facq-120Hz _s-Back_Img-2000**	120	GFRP006	back	1 to 1.8
**GFRP-007_facq-145Hz _s-Front_Img-2000**	145	GFRP007	front	0.2 to 1
**GFRP-007_facq-120Hz _s-Back_Img-2000**	120	GFRP007	back	1 to 1.8
**GFRP-008_facq-145Hz _s-Front_Img-2000**	145	GFRP008	front	0.2 to 1
**GFRP-008_facq-120Hz _s-Back_Img-2000**	120	GFRP008	back	1 to 1.8

## 2 Experimental Design, Materials, and Methods

The pulsed thermography procedure is composed of three main stages: (1) a CRFP/GFRP sample with internal artificial defects is placed perpendicularly to the IR camera at a fixed distance, the power flashes are placed in reflection mode [1] between the camera and the specimen also at a fixed distance, (2) the acquisition parameter (temperature calibration range, emissivity, integration time) are set in the IR acquisition software, and (3) the inspected sample is heated with the thermal stimulus of the heat sources and simultaneously the temperature evolution of its surface is recorded. These stages are shown in Fig 1.

The acquisition procedure was performed in one session under constant conditions for all the tested specimens (Table 1). The dataset was generated with a pulsed thermography experiment (Tables 2 and 3) on three CFRP samples and three GFRP samples. The CFRP006/GFRP006 samples have a flat geometry (Fig. 2a). The CFRP007/GFRP007 and CFRP008/GFRP008 specimens have a curved and trapezoidal geometries, respectively (Fig. 2b and c). Each of these samples contain 25 square internal defects (Teflon inserts) with variable area, and depth, but all having the same thickness.

The infrared camera records for several seconds (approximately 16 to 17 seconds) the thermal evolution on the surface of the inspected CFRP/GFRP sample while applying a heat pulse of 2 ms (at Full-width half-maximum) and 12.8 kJ of energy. Each specimen/sample was tested in reflection mode under two conditions. The first condition takes the thermal images on the front surface from the composite material where the defective zones are close to this side (shallower). A second condition takes the thermal images on the back surface where the defects are deeper.

The acquisition of the database required the below materials.

• **CFRP006 (**Fig. 2**a)**
• **CFRP007 (**Fig. 2**b)**
• **CFRP008 (**Fig. 2**c)**
• **GFRP006 (**Fig.s 2**a)**
• **GFRP007 (**Fig. 2**b)**
• **GFRP008 (**Fig. 2**c)**
• **FLIR IR Camera (**Table 2**)**
• **BALCAR Flashes**
• **FLIR ResearchIR Acquisition software**
• **Sync system (custom-built)**

## CRediT authorship contribution statement

**Jorge Erazo-Aux:** Conceptualization, Data curation, Funding acquisition, Writing - original draft. **Humberto Loaiza-Correa:** Supervision, Writing - review & editing, Resources. **Andres David Restrepo-Giron:** Writing - review & editing. **Clemente Ibarra-Castanedo:** Conceptualization, Methodology, Investigation, Writing - review & editing. **Xavier Maldague:** Supervision, Resources.

## Declaration of Competing Interest

The authors declare that they have no known competing financial interests or personal relationships which have, or could be perceived to have, influenced the work reported in this article.

## Appendix Supplementary materials

Image, application 1
